# Thermally Stable Amorphous Oxide-based Schottky Diodes through Oxygen Vacancy Control at Metal/Oxide Interfaces

**DOI:** 10.1038/s41598-019-44421-x

**Published:** 2019-05-27

**Authors:** Seung-Min Lim, Han-Wool Yeon, Gi-Baek Lee, Min-Gi Jin, Seung-Yong Lee, Janghyun Jo, Miyoung Kim, Young-Chang Joo

**Affiliations:** 1 Department of Materials Science and Engineering, Seoul National University, Seoul, 08826 Republic of Korea; 2 Research Institute of Advanced Materials (RIAM), Seoul National University, Seoul, 08826 Republic of Korea

**Keywords:** Electronic devices, Nanoscience and technology

## Abstract

Amorphous oxide semiconductor (AOS)-based Schottky diodes have been utilized for selectors in crossbar array memories to improve cell-to-cell uniformity with a low-temperature process. However, thermal instability at interfaces between the AOSs and metal electrodes can be a critical issue for the implementation of reliable Schottky diodes. Under post-fabrication annealing, an excessive redox reaction at the ohmic interface can affect the bulk region of the AOSs, inducing an electrical breakdown of the device. Additionally, structural relaxation (SR) of the AOSs can increase the doping concentration at the Schottky interface, which results in a degradation of the rectifying performance. Here, we improved the thermal stability at AOS/metal interfaces by regulating the oxygen vacancy (*V*_O_) concentration at both sides of the contact. For a stable quasi-ohmic contact, a Cu-Mn alloy was introduced instead of a single component reactive metal. As Mn only takes up O in amorphous In-Ga-Zn-O (a-IGZO), excessive *V*_O_ generation in bulk region of a-IGZO can be prevented. At the Schottky interfaces, the barrier characteristics were not degraded by thermal annealing as the Ga concentration in a-IGZO increased. Ga not only reduces the inherent *V*_O_ concentration but also retards SR, thereby suppressing tunneling conduction and enhancing the thermal stability of devices.

## Introduction

To overcome the scaling limits of current memory devices, the integration of crossbar circuit devices^1–3^ has attracted great interest. When each cell is located at the cross point of the interconnects, the density of memory can be both increased by reducing the cell area of the 4F^2^ (F = minimum feature size) and further enhanced by 3D stacking^4,5^. However, for the realization of crossbar circuits, two terminal-based selection devices are also required as well as memory elements to prevent interference from adjacent cells, which is referred to as the cross-talk problem^6,7^. By incorporating highly nonlinear current-voltage (*I–V*) components, such as metal oxide-based Schottky diodes^5,8^ and chalcogenide-based ovonic threshold switches^9,10^, the sneak current in circuits can be regulated.

However, as the downsizing of devices increases, the nonuniform properties of crystalline semiconductors caused by grain boundaries can be an issue in the consideration of cell-to-cell uniformity in crossbar circuits^6,11^. In this respect, amorphous oxide semiconductor (AOS)-based Schottky diodes have been recently developed because of their long-range disorder-related unique properties, enhanced uniform electrical properties and mechanical flexibility with a low-temperature fabrication process^12–15^, which has merits for flexible^16,17^ and large-area applications^18,19^.

The rectifying performance of AOS Schottky diodes has been improved by modifying the interface of the metal/AOS contacts, which increases the effective barrier height at the Schottky interface (*Φ*_B_) and decreases the contact resistance at the ohmic interface^16–18^. However, thermal stability issues in AOS-based Schottky diodes should also be considered as well as their performance since the interface characteristics of metal/AOS contacts are vulnerable to post-annealing processes for the following reasons.

**Figure 1:**
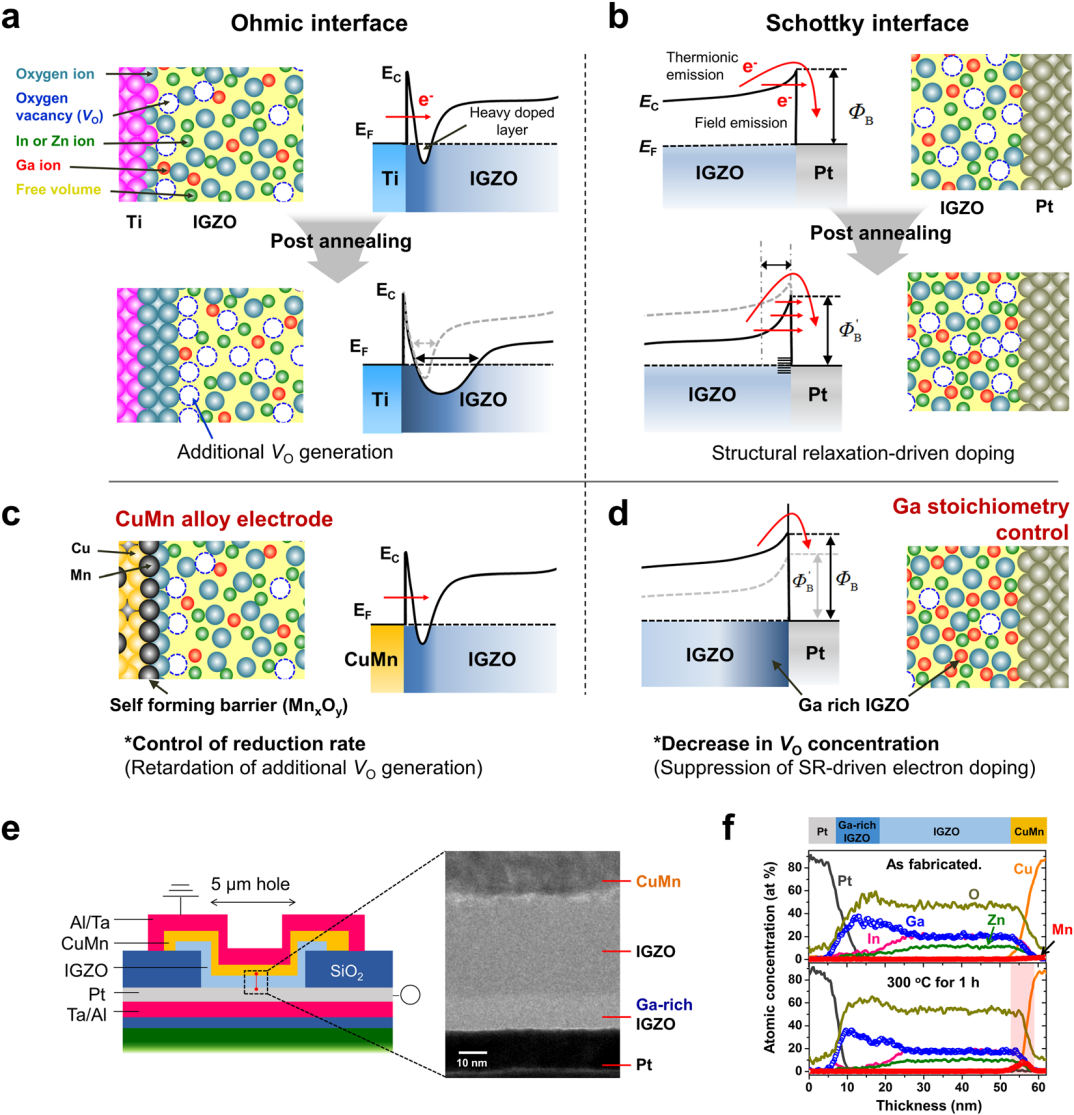
Schematics and energy band diagram of the ohmic and Schottky contacts in AOS-based Schottky diodes. (**a**) A degradation of the Schottky barrier height caused by intrinsic structural relaxation driven doping of the AOS at the Schottky contact. (**b**) Moreover, an excessive interfacial reaction at the ohmic contact can affect the bulk oxide region. The thermal stability of devices can be improved by (**c**) introducing a metal alloy electrode and (**d**) enriching the Ga concentration at the Schottky interface. (**e**) Schematic structure, cross-sectional TEM image of the Schottky diode and following (**f**) the EDS composition profile of devices before and after annealing at 300 °C for 1 h. The corresponding position was marked in a red line at (**e**). After the post-annealing process, the segregation of Mn in CuMn was observed, and high Ga concentration in the vicinity of the Schottky interface was well maintained.

For ohmic contacts, the interfacial reduction of AOSs is induced by using reactive metals (e.g., Ti and Al) that have a strong affinity for oxygen. Reactive metals uptake oxygen from the AOSs and increase the oxygen vacancy (*V*_O_) concentration. Additionally, *n*-type dopants in the interface region of the AOSs (*i.e*., heavily doped interfacial layer) result in tunneling-dominant conduction at the interface (quasi-ohmic contact)^20,21^, as illustrated in Fig. 1a. However, when the interfacial reactions are not regulated, the doping concentration in the bulk region of the AOSs will also be affected by the interfacial reactions, thereby inducing a breakdown of the devices as well as degrading *Φ*_B_.

At the Schottky contact, intrinsic atomic rearrangement in AOSs, which is known as structural relaxation (SR)^22–24^, can affect the rectification property of diodes under thermal circumstances, even below the glass transition temperature (*T*_g_), by increasing the doping concentration of the AOSs without changes in the absolute *V*_O_ concentration. The origin of SR-driven doping suggests that the densification of a local structure with *V*_O_s transforms the electronic state of deep-donor-*V*_O_s to shallow-donor-*V*_O_s^25^. Furthermore, as the atomic mobility at the metal/AOS interface is faster than that in the AOS bulk, SR-driven electron doping may intensively occur at the interface^25^. Thus, even though noble metals (e.g., Pt) can prevent interfacial reduction at the Schottky interface, intrinsic SR-driven doping can lead to tunneling conduction through *V*_O_s in the shallow donor state and a decrease in *Φ*_B_^26^ **(**Fig. 1b**)**. Therefore, to ensure thermal stability in AOS Schottky diodes, controlling the formation of *V*_O_s at each side of the contacts is a prerequisite.

Here, we demonstrate a thermally stable amorphous In-Ga-Zn-O (a-IGZO) Schottky diode by suppressing the excessive reduction of the AOS at the ohmic contact and the SR-driven doping of the AOS at the Schottky interface. For a thermally stable ohmic interface, a Mn-doped Cu electrode was introduced instead of a single component reactive metal **(**Fig. 1c**)**. In a previous study, the application of a CuMn alloy for an ohmic contact on a-IGZO with the elucidation of the ohmic contact formation mechanism was reported^27^. The results indicate that Mn in the CuMn alloy uptakes oxygen from a-IGZO and forms a Mn_x_O_y_ interfacial layer (~5 nm), which generates a heavily doped layer and blocks Cu diffusion into a-IGZO. In this study, we focus on a slow rate of interfacial reduction solely induced by Mn in the CuMn alloy compared to pure reactive metals. As the small amount of Mn (2 at% Mn) uptakes oxygen at the a-IGZO/CuMn interface, excessive interfacial reduction can be suppressed, resulting in a stable quasi-ohmic contact with further thermal annealing up to 300 °C. However, the Ti contact induces the electrical breakdown of a-IGZO diodes, implying that the *V*_O_ concentration in a-IGZO significantly increased after annealing.

On the Schottky contact side, the Ga stoichiometry of a-IGZO was controlled to suppress the tunneling conduction through the *V*_O_s in the shallow donor state, as illustrated in Fig. 1d. Ga is known as a suppressor of *V*_O_ formation in a-IGZO due to the stronger oxygen bonding energy of Ga compared with that of In or Zn. The Ga-O bonding energy in a-IGZO was reported to be 2.0 eV, which is higher than that of In-O (1.7 eV) or Zn-O (1.5 eV)^28^. Our expectation is that increasing the Ga concentration in a-IGZO will decrease the concentration of the *V*_O_s in the shallow donor state and deep donor state. Thus, the impact of SR-driven electron doping will be reduced as the Ga concentration increases. As a result, tunneling conduction will be suppressed at both the initial state and the relaxed state, thereby maintaining an effective Schottky barrier height after SR. Our a-IGZO diodes show that the thermal stability of Schottky barrier characteristics increases as the Ga concentration increases.

Interestingly, we also found that as the Ga concentration increased, the degree of SR (*i.e*., degree of densification of the a-IGZO film) decreased using a thin-film curvature measurement system. An increased average bonding energy of Ga-rich a-IGZO is be strongly related to this phenomenon, although further study is needed to identify the exact role of Ga in the structural rearrangement of a-IGZO. We believe that the discovered rationale in this work, the regulation of *V*_O_-related interfacial reactions at a-IGZO/metal contacts via metal alloys and Ga stoichiometry engineering can be utilized as a guideline for the demonstration of thermally stable amorphous oxide electronic devices.

## Result and Discussion

### Design of a-IGZO Schottky diodes

Figure 1e shows schematics of Schottky diodes composed of CuMn/a-IGZO/Pt films with a Ta/Al passivation layer. In the cross-sectional transmission electron microscopy (TEM) image of the device, 10-nm-thick Ga stoichiometry-controlled a-IGZO films are clearly shown. After annealing at 300 °C for 1 h, the concentration profiles of the elements in the devices were nearly unchanged except for Mn, as shown in the TEM-energy dispersive spectroscopy (EDS) results in Fig. 1f. The Mn concentration locally increased at the CuMn/a-IGZO interface from 2 at% to 8.2 at% after annealing, whereas the Cu concentration was nearly maintained. These results are similar to the previous result^27^ that a Mn-rich oxide layer is formed at the a-IGZO/CuMn interface. This oxide layer induces a heavily doped layer for the quasi-ohmic contact that blocks Cu diffusion that was detrimental to the stability of a-IGZO electronics because of the electrochemical reactivity of Cu in a-IGZO^29^.

### Thermally stable ohmic contact with Cu-Mn electrode

**Figure 2:**
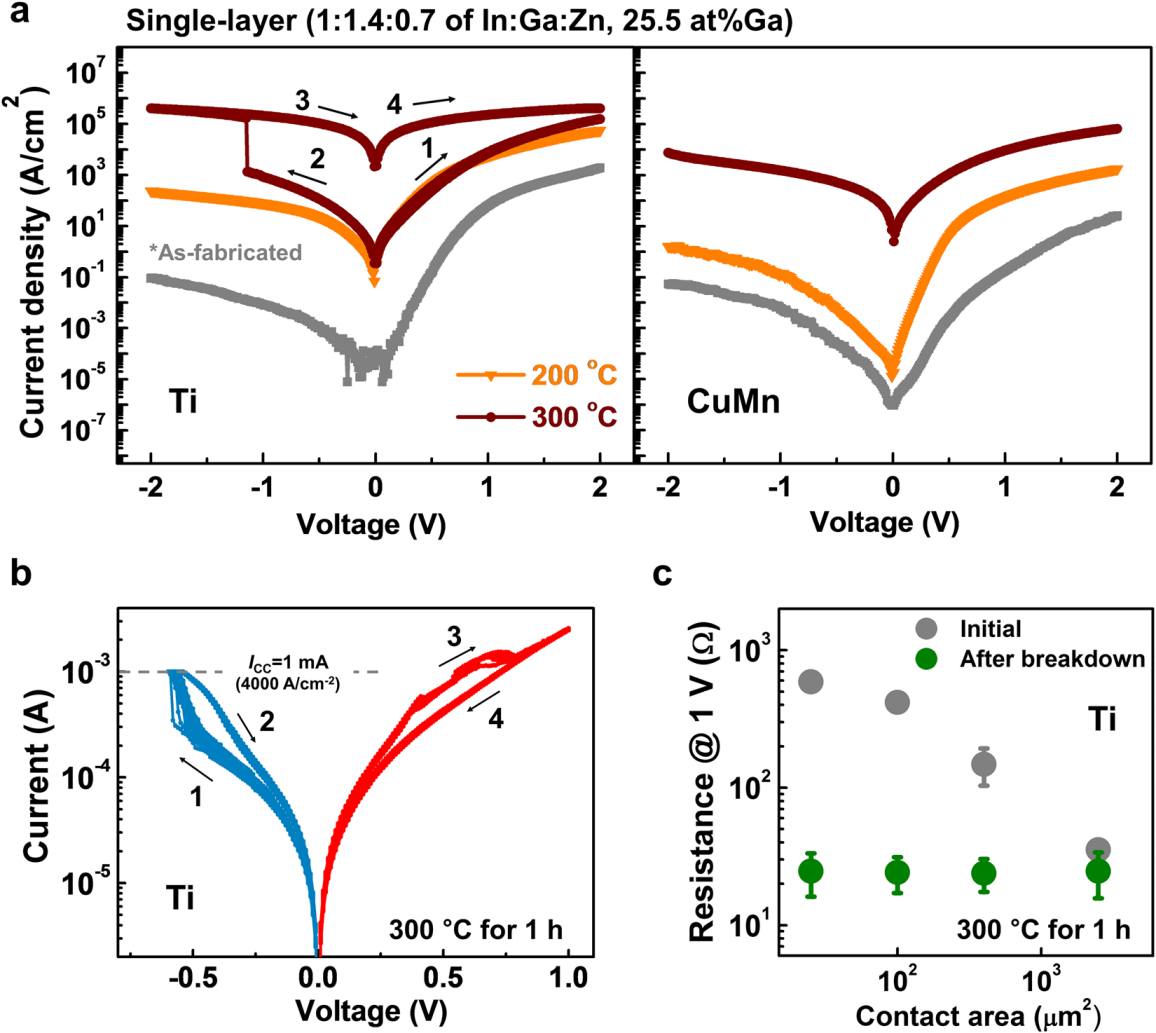
Representative *I–V* curves of the Ti diodes and the CuMn diodes with respect to the annealing temperature. (Annealing time was fixed for 1 h). (**a**) As the annealing temperature increases, the electrical conductance of the devices typically increases. However, after annealing at 300 °C, the Ti diodes are electrically broken down under reverse bias application, whereas the CuMn diodes still show rectification behavior with degraded performance compared to diodes annealed at 200 °C. (**b**) Bipolar resistive switching behavior with the compliance current (~1 mA) when the negative voltage is biased. (**c**) Changes in the resistance (at 1 V) of the Ti diodes before (gray circle) and after breakdown (green circle) with regard to the contact area. The contact area was varied from 25 µm^2^ to 2500 µm^2^.

Figure 2a presents the semilogarithmic *I–V* curves of the Schottky diodes with respect to the ohmic contact materials and annealing temperature. To focus on the effect of the ohmic contact materials, single-layered a-IGZO (In:Ga:Zn = 1:1.4:0.7) was considered. In the as-fabricated state, Ti diodes exhibited a higher forward current density than the CuMn diodes; however, they have a similar conductance under reverse bias conditions. When the Schottky conduction parameters, *Φ*_B_, the ideality factor (*n*), and the series resistance (*R*_S_), are compared, the *R*_S_ of the Ti diodes is markedly lower than that of the CuMn diodes (Fig. S1, Supplementary information). As the *R*_S_ of thin-film-type Schottky diodes is governed by the contact resistance of the ohmic contact resistance, this result indicates that the ohmic contact resistance of Ti is lower than that of CuMn. It can be elucidated that pure Ti will take oxygen from a-IGZO with more than 2 at% Mn at its initial state, which leads to the formation of a lower quasi-ohmic contact resistance. After annealing at 200 °C, although the forward current density of Ti diodes increased, the rectifying behavior of Ti diodes became weak as the reverse current drastically increased. On the other hand, CuMn diodes exhibited an enhanced forward/reverse current ratio (F/R ratio) with a low level of *n*. These results imply that an additional reduction at the Ti/a-IGZO interface significantly degraded the rectifying performance of the diodes, whereas the CuMn/a-IGZO interface regulated the excessive interfacial reduction due to the relatively slow reduction rate.

As the annealing temperature increased to 300 °C, the rectifying performance of both Ti and CuMn diodes was diminished. Interestingly, Ti diodes showed electrical breakdown under the negative bias condition, whereas a hysterical *I–V* curve was not observed in the CuMn diodes. Furthermore, when the compliance current density was set at 4 kA cm^−2^, reversible electrical breakdown behavior (*i.e*., resistive switching) was observed, as shown in Fig. 2b.

The resistance of the devices became independent of the contact area of the devices, whereas area-dependent resistance changes were measured at the initial state (Fig. 2c). These results strongly suggest that the interfacial reduction at the Ti/a-IGZO interface additionally forms *V*_O_s in a-IGZO and that the concentration of mobile *V*_O_s becomes high enough to become localized, which leads to reversible conduction channels in a-IGZO driven by O vacancy drift toward the Pt bottom electrodes^30^. However, the CuMn electrodes can suppress the generation of excess *V*_O_s because of the kinetical regulation (*i.e*., a small amount of Mn as a reducing agent) of interfacial reduction. Notably, although excessive interfacial reduction was suppressed, the rectifying performance of the CuMn diodes deteriorated. As shown in Fig. S1, a decrease in *Φ*_B_ (0.76 eV → 0.31 eV) with increasing *n* (1.6 → 4.7) implies that tunneling conduction through the Schottky barrier became significant. These phenomena can be elucidated as follows: SR-driven electron doping in a-IGZO increased the space charge density, composed of shallow donor state *V*_O_s, which increased in the vicinity of the Schottky contact, resulting in a decrease in the effective Schottky barrier height^21^.

Therefore, it is important to improve the thermal stability of the Schottky barrier at the a-IGZO/Pt interface to maintain high rectification performance. Among In, Ga, and Zn, Ga suppresses *V*_O_ formation in a-IGZO because Ga showed the highest Gibbs free energy of oxidation^31^ (thermodynamic aspect) and formed the strongest oxide bond^28^ (kinetic aspect). Thus, it is expected that SR-driven doping, originating from the *V*_O_s_,_ is regulated in Ga-rich a-IGZO^32^. Note that increasing the Ga concentration of a-IGZO also leads to an increase in the bulk resistance of devices, which induced a decrease in the forward current density with poor rectifying performance. To decrease the bulk resistance of a-IGZO as well as suppress the SR at the Schottky interface, the Ga concentration was partially increased, i.e., 10 nm among the 50 nm-thick a-IGZO film near the Pt interface by co-sputtering with Ga_2_O_3_

### Effect of Ga-stoichiometry control of a-IGZO at Schottky interfaces

**Figure 3:**
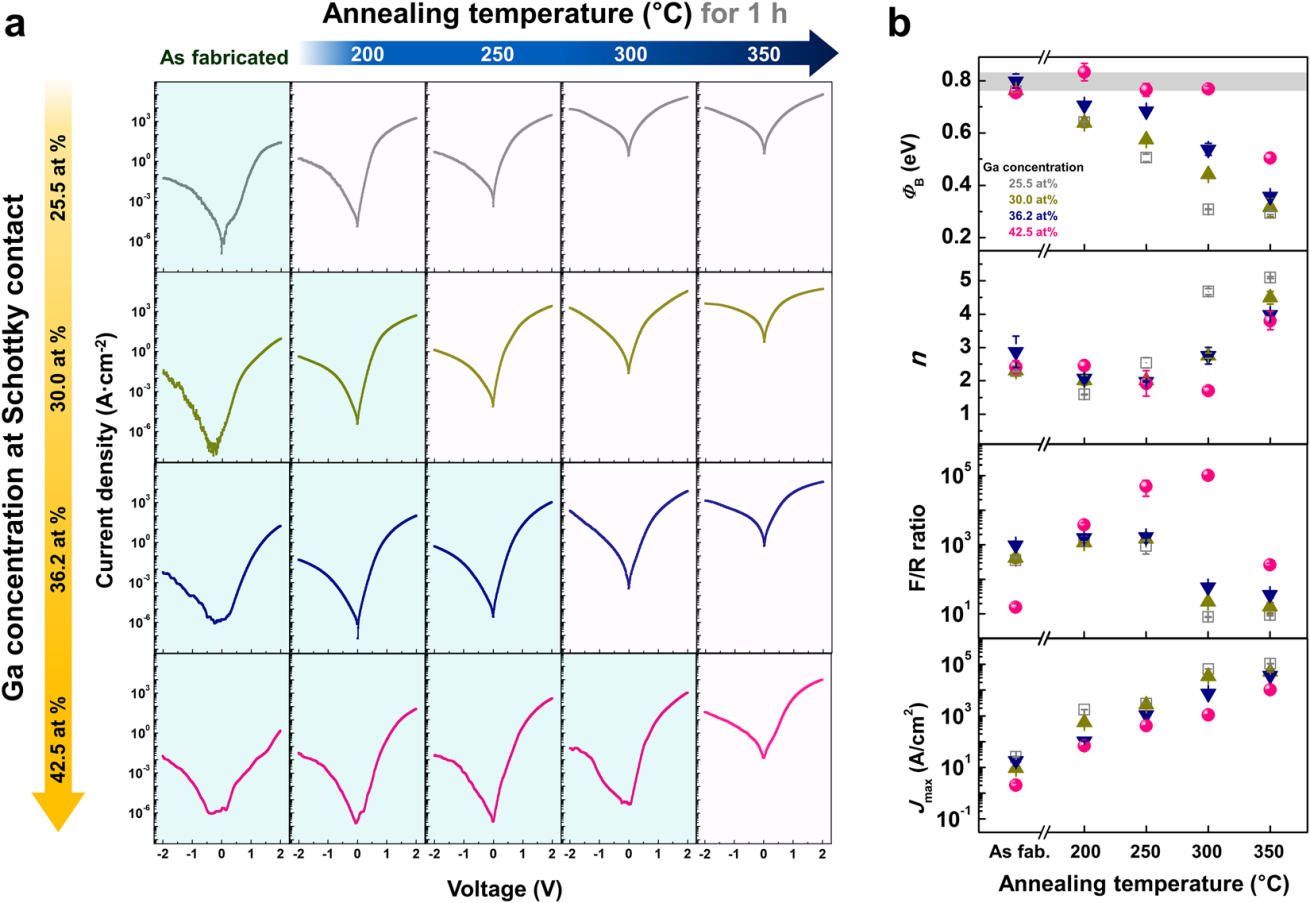
Electrical characterization of a-IGZO diodes with Ga-rich layer at Schottky contact. (**a**) *I–V* characteristics and (**b**) following diode parameters of the CuMn diodes considering both the annealing temperature from 200 °C to 350 °C and the Ga concentration in a-IGZO in the vicinity of Pt interface from 25.5 to 42.5 at%. When the ratio of the stabilizing Ga cation at the Schottky interface increases, the reverse current density was well maintained after the post-fabrication annealing process, which was verified by the behavior of the Schottky barrier height and ideality factor.

Figure 3a presents the *I–V* characteristics of the CuMn diodes composed of Ga stoichiometry-controlled a-IGZO at the Schottky contact with respect to the post-fabrication annealing temperature. The stoichiometry of a-IGZO (In:Ga:Zn) at the Pt/a-IGZO interface was modulated from 1:1.4:0.7 (25.5 at% Ga) to 1:9.1:0.7 (42.5 at% Ga), and the annealing temperature varied from 200 °C to 350 °C. In the CuMn diodes with single-layered a-IGZO (25.5 at% Ga), the reverse current rapidly increases with increasing annealing temperature. However, when the concentration of Ga in the Schottky contact region increased, the extent of the reverse current changes with annealing gradually decreased. As the stoichiometry of Ga reached 42.5 at%, the reverse current of the CuMn diodes was nearly unchanged until a 300 °C annealing temperature was attained.

From the diode parameters derived from the *I–V* characteristics based on the Schottky conduction mechanism (Fig. 3b), the change in *Φ*_B_ and *n* with the post-fabrication annealing process was effectively controlled by introducing the Ga-rich interlayer at the Schottky contact. In particular, when the Ga concentration at Schottky contact increased to 42.5% Ga, *Φ*_B_ (~0.76 eV) and *n* (~2.5) remained unchanged until an annealing temperature of 300 °C. This phenomenon could be clear evidence that increasing the Ga concentration in the vicinity of the Schottky contact is effective for regulating SR-driven electron doping by suppressing *V*_*O*_s, thus enhancing the thermal stability of devices. As the post-fabrication annealing temperature increases, *J*_*max*_ significantly increases due to the formation of the quasi-ohmic contact between CuMn and a-IGZO^27^. Although a partial increase in Ga concentration at the Schottky contact causes a slight resistance increase in the oxide, which leads to a reduction in forward current density. The F/R ratio notably increases 10^4^ times after annealing at 300 °C since controlling the reverse current with annealing is much more effective on the overall rectification property of the diodes. From a comparison of the *I–V* characteristics and following the Schottky diode parameters of various diodes, we can achieve a reliable F/R ratio of 1.0 × 10^5^ and a *J*_max_ of 1.1 × 10^3^ A/cm^2^ until 300 °C annealing is reached. After 350 °C annealing, the forward current level of the Cu diodes increases further. However, the rectification behavior of whole device was deteriorated by SR-driven electrical doping in the bulk region of a-IGZO. This finding can be confirmed by the sharp change in *Φ*_B_ and *n* between 300 °C and 350 °C annealing.

### Mechanical analysis for structural changes of a-IGZO with different Ga ratio

**Figure 4:**
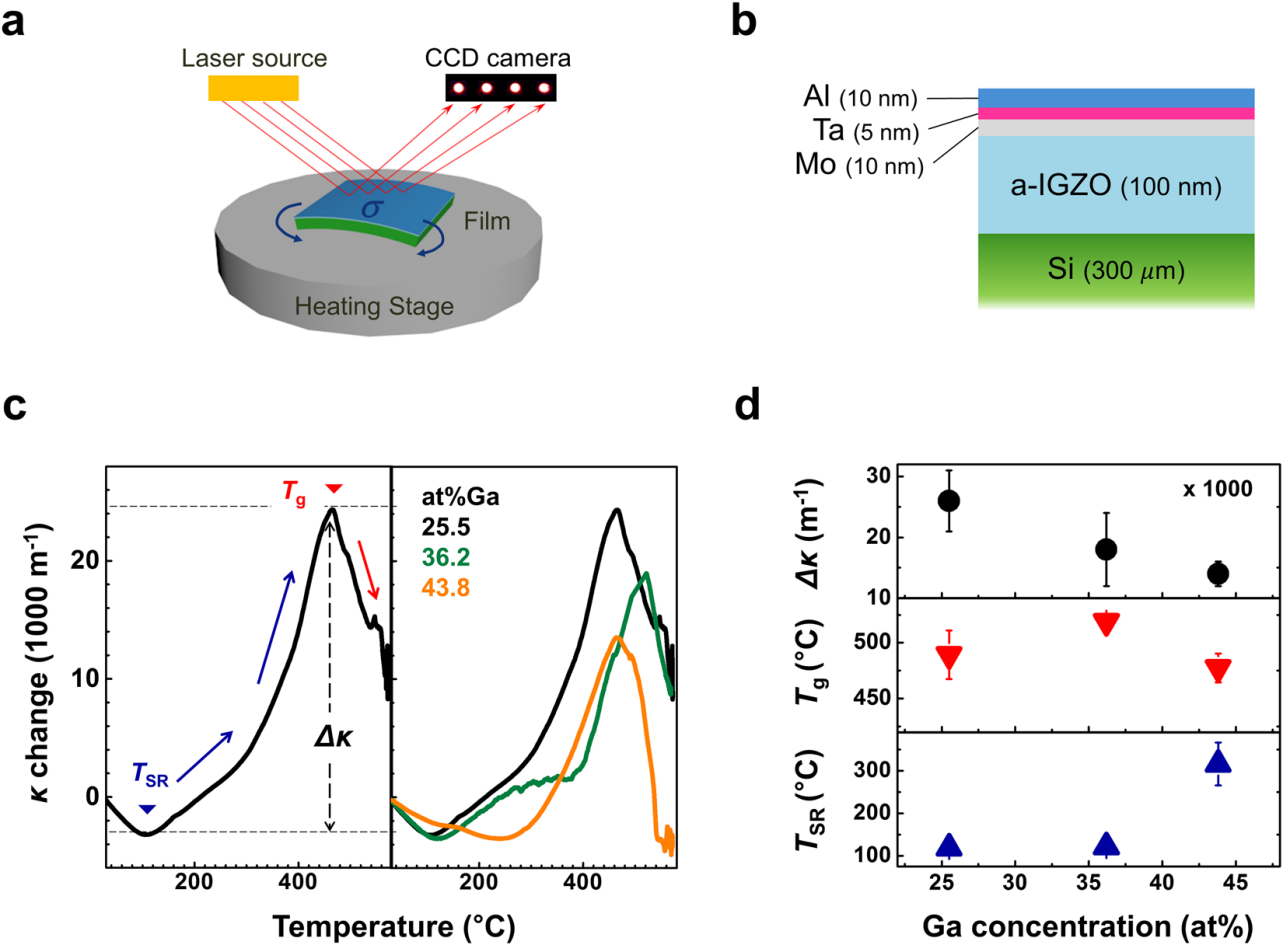
Mechanical analysis for structural changes of a-IGZO with respect to Ga concentration. (**a**) Schematic illustrations of *In situ* wafer curvature measurement system using a multibeam optical sensor. (**b**) Schematic cross-sectional images of multilayered thin films for curvature measurement. An additional Al/Ta/Mo layer was deposited for encapsulation. (**b**) Curvature changes in a-IGZO thin films during the heat cycle and (**c**) measured *T*_SR_, *T*g and maximum curvature change (Δκ) as a function of the Ga concentration in a-IGZO.

To verify the effect of Ga stoichiometry control on the SR behavior of a-IGZO, an *in situ* wafer curvature measurement system^22,33^ was introduced. (Fig. 4a). By monitoring curvature changes with the volumetric changes of the a-IGZO films by SR and glass transition during heating, the parameters describing structural and phase changes in amorphous materials can be precisely quantified^22^. The detailed information for analysis method of curvature measurement system was provided in Fig. S2 (Supplementary information). In this work, multilayer thin films composed of Al/Ta/Mo/a-IGZO on a Si wafer were used to prevent extrinsic effects on a-IGZO during heating (Figs 4b and S2c).

The left side of Fig. 4c shows the representative curvature change characteristics of a-IGZO films with 25.5 at% Ga. The positive and negative curvature changes are attributed to the tensile and compressive stresses applied to the Si wafer caused by the volume shrinkage and expansion of a-IGZO during the structural changes, respectively. After the slight decrease in the curvature by thermal expansion of a-IGZO, a transition in the curvature change behavior was observed at 120 °C, which indicates the initiation of SR of a-IGZO. The wafer curvature continuously changes in the positive direction by the SR-driven volume shrinkage of a-IGZO. After 480 °C, the wafer curvature changes in the negative direction due to the glass transition of a-IGZO.

The right side of Fig. 4c exhibits the curvature change behavior of a-IGZO-based films with respect to Ga concentration in a-IGZO. The parameters indicating a structural change in a-IGZO, such as the onset temperature of SR (*T*_SR_), glass transition temperature (*T*g) and maximum curvature change (Δκ), were extracted from curvature-temperature curves and are presented in Fig. 4d. When the Ga concentration in a-IGZO increased to 42.5 at%, *T*_SR_ significantly increased to 315 °C, and a decrease in Δκ was also observed during SR. These results indicate that the SR of a-IGZO can be effectively retarded by Ga stoichiometry control in a-IGZO. Along with the mechanical analysis for investigating changes in SR behavior, the *I–V* characteristics of a-IGZO were analyzed under various annealing conditions with respect to Ga concentration, as shown in Fig. S3 (Supplementary information).

### Thermal stability enhancement of interfacial controlled a-IGZO Schottky diodes

**Figure 5:**
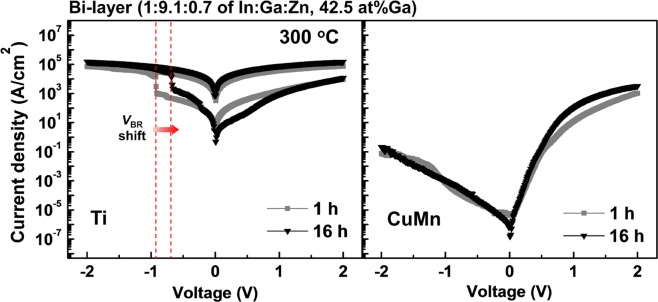
*I–V* characteristics of Ga-rich a-IGZO diodes after annealing at 300 °C with increasing time. Until 16 h of annealing was reached, the rectifying performance of the CuMn diodes was barely changed, whereas the electrical breakdown was shifted ahead in the case of the Ti diodes.

To confirm the thermal stability of the optimized devices, the *I–V* characteristics of Ga-rich a-IGZO diodes were measured by varying the post-annealing process time from 1 h to 16 h (Fig. 5). The annealing temperature and Ga concentration at the Schottky interface were fixed at 300 °C and 42.5 at% Ga. In the reference Ga-rich Ti diodes, the electrical breakdown of the devices was observed during the negative bias sweep, as already discussed in Fig. 2a. Moreover, the breakdown voltage decreased from −0.98 V to −0.67 V when the annealing time increased. This finding can be inferred by the fact that the extent of the interfacial reaction at the ohmic interface is closely connected to the diffusion of ions in the metal oxide, which is dependent on the annealing time. On the other hand, the *I–V* characteristics of Ga-rich CuMn diodes were nearly unchanged regardless of the post-fabrication annealing conditions. These results strongly suggest that SR-driven doping in amorphous oxides can be regulated by the incorporation of a *V*_O_ suppressor.

From the above results, it was demonstrated that the thermal stability of a-IGZO-based Schottky diodes as well as their rectifying performance can be enhanced by regulating the interfacial reduction reaction and SR-driven atomic rearrangement in a-IGZO under additional thermal circumstances. Our findings on *V*_O_ control at the metal/oxide contacts provide a strategy for an improvement in reliability issues in AOS-based electronic devices.

## Discussion

We have developed a thermally stable a-IGZO Schottky diode through both the regulation of the interface reduction reaction at the ohmic contact and the SR-driven electron doping of a-IGZO at the Schottky contact. The CuMn (2 at% Mn) alloy forms a thermally stable ohmic contact because only the small amount of Mn induces the interfacial reduction of a-IGZO during post-fabrication annealing and suppresses excessive *V*_O_ generation in a-IGZO, which affects the bulk resistance. However, Ti induces the electrical breakdown of a-IGZO caused by *V*_O_-based localized conduction channels. For the thermally stable Schottky contact, a 10-nm-thick Ga-rich a-IGZO layer was introduced at the Schottky interface. When the Ga concentration was 25.5 at% (In:Ga:Zn = 1:1.4:0.7) without a Ga-rich interlayer, the rectifying performance deteriorated after annealing because SR increased the shallow donor state *V*_O_ concentration of a-IGZO and tunneling through the Schottky barrier became significant. However, the Ga-rich interlayer (42.5 at% Ga, In:Ga:Zn = 1:9.1:0.7) renders thermally stable Schottky barrier characteristics, as Ga decreased the *V*_O_ concentration, the origin of SR-driven electron doping. Furthermore, the degree of SR was reduced in Ga-rich a-IGZO, which also suppresses SR-driven electron doping. The a-IGZO diodes based on interfacial *V*_O_ engineering points to future opportunities in thermally stable AOS Schottky diodes with desirable rectifying performance.

## Methods

### Fabrication of a-IGZO Schottky diode

A 30-nm-thick Pt layer was deposited onto a Ta/Al adhesive layer-coated SiO_2_ (100 nm)/Si wafer by e-beam evaporation as the Schottky contact, bottom electrode (BE) of the devices. A SiO_2_ isolation layer (300 nm) was deposited onto a platinized substrate by plasma-enhanced chemical vapor deposition at a substrate temperature of 300 °C. Square holes ranging from 25 to 10,000 µm^2^ were patterned in the isolation layer using photolithography and dry etching. Afterwards, the hole patterns were filled with a-IGZO thin films by radio frequency (RF) sputtering at room temperature. For the 10-nm-thick a-IGZO bottom layer, the Ga concentration was modulated via co-sputtering of the InGaZnO_4_ and Ga_2_O_3_ targets. The 40-nm-thick a-IGZO layers were subsequently deposited without co-sputtering. The deposition process was performed under a working pressure of 5 mTorr with gas flow rates for argon and oxygen of nineteen and one standard cubic centimeter per minute, respectively. The stoichiometry of a-IGZO was measured by X-ray photoelectron spectroscopy (XPS, PHI 5000 VersaProbe). The thin films of a-IGZO were patterned as 65 µm × 65 µm square patches using photolithography and hydrofluoric acid (HF) etching, and the patches were covered with CuMn (30 nm) or Ti (30 nm) by direct current (DC) magnetron sputtering (ULTECH) for the ohmic contact, top electrode (TE). The Ta/Al bilayer (50/50 nm) was subsequently deposited on the TE to prevent external reactions with the ambient atmosphere during the annealing process.

### Device characterization

The fabricated devices were annealed at 200–350 °C for 1 h in high vacuum (10^−6^ Torr). After annealing, the changes in the current-voltage (*I–V*) characteristics of the devices were measured using the Agilent 4156 C parameter analyzer at room temperature. A voltage was applied to the BE, while the TE was grounded. The physical structures and compositional profile of the devices were investigated by TEM (JEOL, JEM-2100F) and EDS (Oxford instruments) analysis.

### *In situ* wafer curvature measurements

To investigate the structural changes in a-IGZO, the curvature change in a-IGZO-based films was monitored during the heating process using a multibeam optical sensor (*k*-Space Associates, Inc.). For the passivation of a-IGZO films, an Al/Ta/Mo (10/5/10 nm) layer was deposited in sequence by DC magnetron sputtering and loaded onto the heating stage in the chamber. The passivated a-IGZO films were heated until 575 °C, where the heating rate was set to 5 °C /min. The working pressure in the chamber was fixed to 10 Torr in N_2_ to prevent air convection at high temperature.

## Supplementary information

Supplementary Information

## Data Availability

The data that support the findings of this study are available from the corresponding author on reasonable request.
