# Supplementary material for: Thermally Stable Amorphous Oxide-based Schottky Diodes through Oxygen Vacancy Control at Metal/Oxide Interfaces
Source: Sci Rep. 2019 May 27;9:7872. doi: 10.1038/s41598-019-44421-x (PMC6536494; doi:10.1038/s41598-019-44421-x)
Supplement: Supplementary file 1 — Supplementary Information [file 41598_2019_44421_MOESM1_ESM.docx]

Supplementary information

**Thermally Stable Amorphous Oxide-based Schottky Diodes through Oxygen Vacancy Control at Metal/Oxide Interfaces**

Seung-Min Lim^1,§^, Han-Wool Yeon^1,§^, Gi-Baek Lee^1^, Min-Gi Jin^1^, Seung-Yong Lee^1^, Janghyun Jo^1,2^, Miyoung Kim^1,2^, & Young-Chang Joo^1,2,*^

^1^Department of Materials Science and Engineering, Seoul National University, Seoul, 08826, Republic of Korea

^2^Research Institute of Advanced Materials (RIAM), Seoul National University, Seoul, 08826, Republic of Korea

^§^These authors contributed equally to this work.

**Corresponding Author**

Young-Chang Joo ([ycjoo@snu.ac.kr](mailto:ycjoo@snu.ac.kr), Tel.: +82-2-880-8986)

**Calculation of the diode parameters by the thermionic current-voltage expression**

The *I-V* characteristics in the forward bias region with $V>3k_{B}T/q$ are given as^1,2^

$$I_{\mathrm{FORWARD}}=I_{s}\exp\left( \frac{qV-I_{\mathrm{FORWARD}}R_{S}}{nk_{B}T} \right)$$

where $I_{s}$ is the saturation current, $n$ is the ideality factor, and $R_{S}$is the series resistance of the devices. $I_{s}$ can be expressed as^1,2^

$$I_{s}={AA}^{*}T^{2}\exp\left( -\frac{\Phi_{B}}{k_{B}T} \right)$$

where $A$ is the contact area and $A^{*}$ is the effective Richardson constant (theoretically *A**=40.8 Acm^-2^K^-2^ for a-IGZO^3^). As *I*­_S_ determined by extrapolating the semilogarithmic *I* versus *V* curve to *V* = 0, $\Phi_{B}$ can be calculated.

The parameters n and R_S_ can also be obtained from the diode conductance (g_d_), which is written by^4^

$$g_{d}=\frac{dI}{dV}=\frac{qI(1-R_{s}g_{d})}{nkT}$$

It gives

$$\frac{1}{g_{d}}=\frac{nkT}{q}+IR_{s}$$

which has an intercept at I=0 of nkT/q and a slope *Rs* for the plot of *I*/*g*_d_ versus *I.*

**The Schottky diode parameter of single-layered a-IGZO-based diodes**


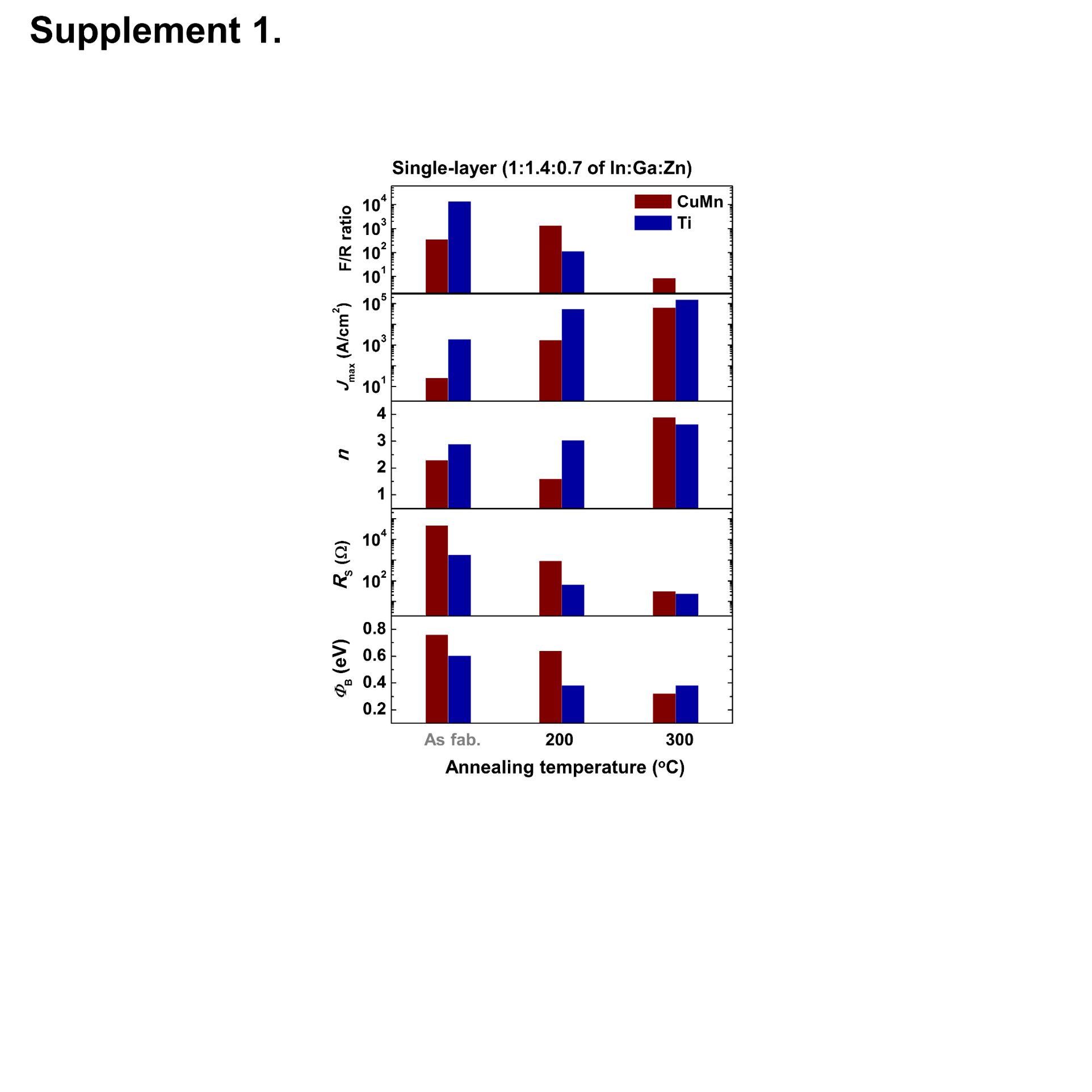


**Figure S1.** The change in the F/R ratio (at $\pm$1.5 V), forward current density at 2 V (*J*_max_) Schottky barrier height ($\Phi$_B_), ideality factor (*n*) and series resistance (*R*_S_) of the CuMn and Ti diodes (single-layered a-IGZO) with different annealing temperatures. The annealing time was fixed to 1 h.

**Wafer curvature measurement system by using multi-beam optical sensor**


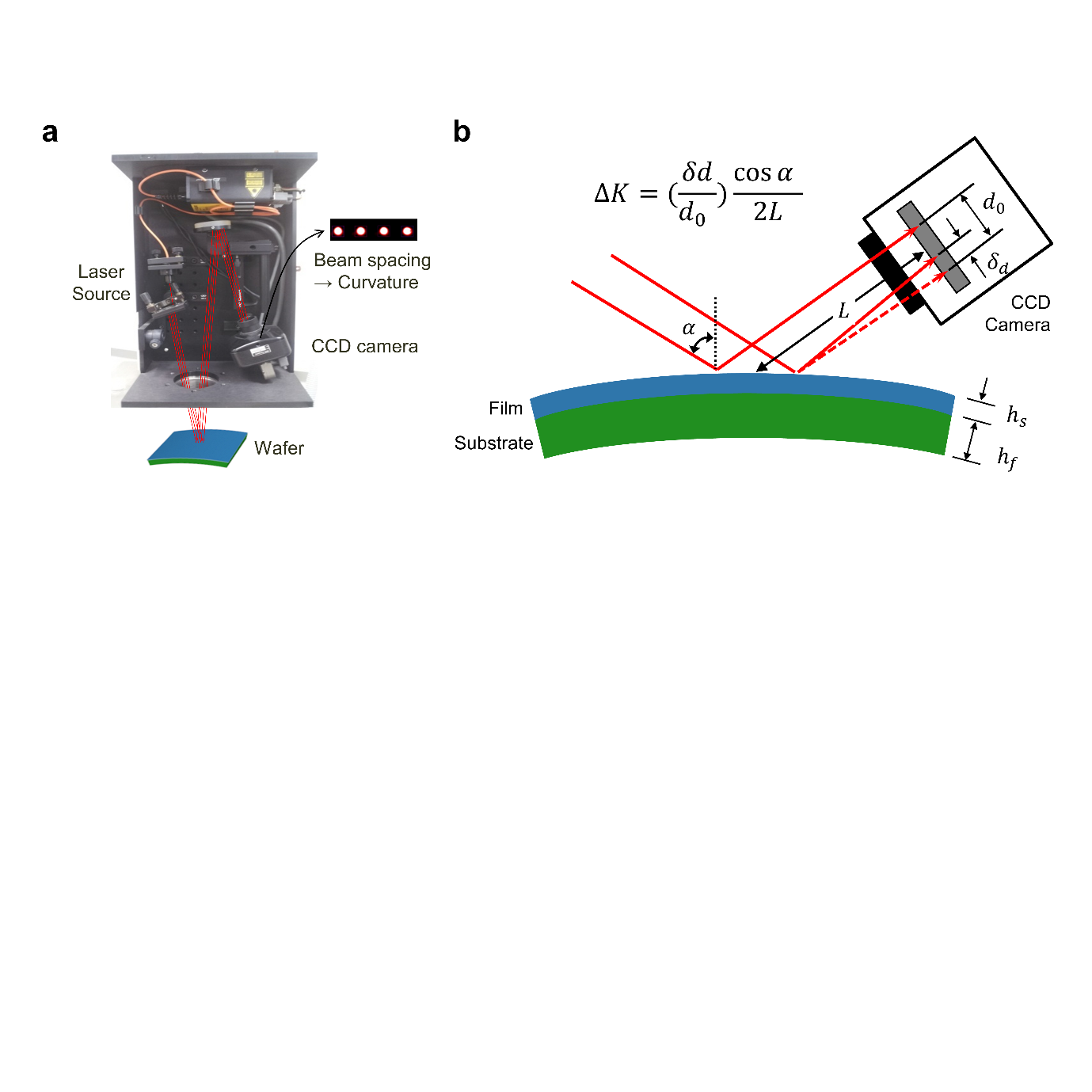


**Figure S2.** (a) Device configuration and (b) illustrations of wafer curvature measurement system using a multi-beam optical sensor. An array of parallel beams irradiates the bended substrate is reflected and the reflected beams are detected by a CCD camera.

To measure the volumetric changes of a-IGZO films induced by the structural relaxations, a wafer curvature measurement system^5,6^ was introduced by using a multi-beam optical sensor. **Fig. S2** shows the operating principle of the wafer curvature measurement systems. An array of parallel laser beams illuminates the film on the bended substrates and the spacing of the reflected beams are detected by a CCD camera. The change in curvature of the substrate ($\Delta K)$ can be obtained from the spacing of the parallel beams reflected from the film ($d$) by the following equations:

$$\Delta K=(\frac{\delta d\text{ }}{d_{0}\text{ }})\frac{\cos\alpha\text{ }}{2L}$$

Where $L$ is the distance between the CCD camera and the wafer, $\alpha$ is the angel between the beam and the normal direction to the film, $d_{0}$ is the initial spacing of the reflected beams.

**Changes in the I-V characteristics with Ga stoichiometric control in a-IGZO**

**
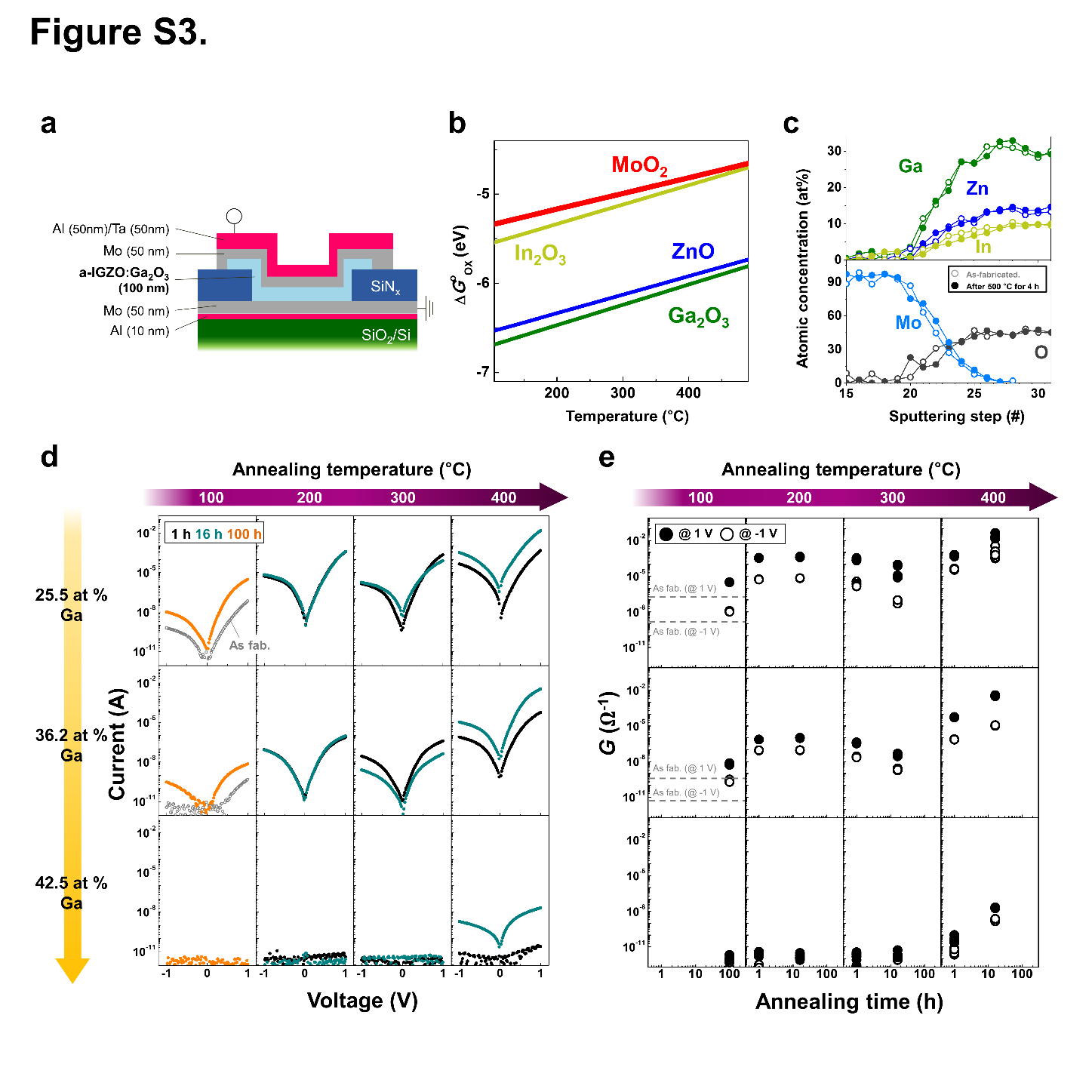
**

**Figure S3.** (a) Schematic structure of the Mo/a-IGZO/Mo devices. (b) Standard Gibbs free energy of Mo, In, Zn and Ga for oxide formation. (c) XPS analysis of the Mo/a-IGZO interfaces with respect to the post-fabrication annealing. (d) Representative *I-V* curve map and (e) conductance (G, at $\pm$1 V) of devices with different Ga concentrations of a-IGZO and annealing conditions. (The contact area was fixed to 25 $\mu$m^2^.) The annealing temperature and time were varied from 100~ 400 °C and 1~ 4 h, respectively.

The changes in the electrical properties of the Mo/a-IGZO/Mo devices with respect to the Ga concentration in a-IGZO were measured. **Fig. S3a** presents the cross-sectional schematic structure of the Mo/a-IGZO/Mo devices. As Mo has a sufficiently high melting temperature^7^ and its Gibbs free energy for oxide formation is higher than that of In, Ga and Zn^8^ **(Fig. S3b)**, the interfacial reaction and interdiffusion to a-IGZO was effectively prevented, as shown in **Fig. S3c**. Therefore, intrinsic changes in the electrical properties of a-IGZO can be observed without external reactions.

**Fig. S3d** shows the *I-V* characteristics map of various diodes considering the concentration of Ga in a-IGZO and post-fabrication annealing conditions. The annealing temperature and time were varied from 100 °C to 400 °C and 1 h to 100 h, respectively. The conductance (G) of devices at $\pm$1 V is shown in **Fig. S3e**. Despite the symmetric structure of the devices, the asymmetric behavior of the *I-V* characteristics was observed. This behavior was induced by the generation of interfacial states during the deposition of the top electrode (TE), which can decrease the effective Schottky barrier height of the TE/a-IGZO devices, as reported in previous studies^9,10^.

In the as-fabricated state, the G of devices decreased as the Ga concentration in a-IGZO increased because Ga suppressed *V*_O_ formation in a-IGZO due to the stronger oxygen affinity than In or Zn. In the case of 25.5 at% Ga, the G of the devices continuously increased as the annealing temperature increased. Additionally, the asymmetry of the *I-V* curve gradually decreased, and nearly symmetric *I-V* characteristic behavior was observed at a 400 °C annealing temperature, induced by the SR-driven doping of a-IGZO.

However, when the Ga concentration in a-IGZO was controlled to 42.5 at%, the G of the devices remained nearly unchanged until 300 °C, even with an increase in the annealing time. At an annealing temperature of 400 °C for 1 h, the *G* of the devices slightly increased, and SR-driven electrical property changes in the *I-V* characteristics were observed after 16 h of annealing. These results indicate that Ga stoichiometric control in a-IGZO can retard SR-driven doping as well as decrease *V*_O_, which is effective in improving the thermal stability of the devices.

**References**

1 S. M. Sze, D. C. M. *Physics of Semiconductor Devices*. 2nd edn, (John Willey & Sons, Inc., 1981).

2 Brillson, L. J. & Lu, Y. ZnO Schottky barriers and Ohmic contacts. *Journal of Applied Physics* **109**, doi:10.1063/1.3581173 (2011).

3 Fung, T.-C. *et al.* Two-dimensional numerical simulation of radio frequency sputter amorphous In–Ga–Zn–O thin-film transistors. *Journal of Applied Physics* **106**, doi:10.1063/1.3234400 (2009).

4 Schroder, D. K. *Semiconductor material and device characterization*. 3rd edn, (Wiley-IEEE Press, 2006).

5 Cho, J. Y., Yang, T. Y., Park, Y. J., Lee, Y. Y. & Joo, Y. C. Structural Instability in Amorphous In-Ga-Zn-O Films Investigated by Mechanical Stress Analysis. *ECS Solid State Letters* **3**, P73-P76, doi:10.1149/2.004406ssl (2014).

6 Cho, J.-Y. *et al.* The phase-change kinetics of amorphous Ge2Sb2Te5 and device characteristics investigated by thin-film mechanics. *Acta Materialia* **94**, 143-151, doi:10.1016/j.actamat.2015.04.058 (2015).

7 Callister, W. D. *Materials science and engineering : an introduction*. 7th edn, (John Wiley & Sons, 2007).

8 I. Barin, K., O. Kubascheski. *Thermochemical properties of inorganic substances*. (Springer, 1973).

9 Yeon, H.-W. *et al.* Structural-relaxation-driven electron doping of amorphous oxide semiconductors by increasing the concentration of oxygen vacancies in shallow-donor states. *NPG Asia Materials* **8**, e250-e250, doi:10.1038/am.2016.11 (2016).

10 Yeon, H.-W. *et al.* Cu Diffusion-Driven Dynamic Modulation of the Electrical Properties of Amorphous Oxide Semiconductors. *Advanced Functional Materials* **27**, doi:10.1002/adfm.201700336 (2017).
